# Global mental health and its social determinants: How should we intervene?

**DOI:** 10.1016/j.brat.2023.104402

**Published:** 2023-10

**Authors:** Crick Lund

**Affiliations:** a Centre for Global Mental Health, Health Service and Population Research Department, Institute of Psychiatry, Psychology and Neuroscience, King's College London, United Kingdom; b Alan J Flisher Centre for Public Mental Health, Department of Psychiatry and Mental Health, University of Cape Town, Cape Town, South Africa

## Abstract

This paper makes the case for expanding the field of global mental health to give more attention to the social determinants of mental health. It does so by describing challenges and opportunities for intervening to address these social determinants, and by presenting some potential approaches to the choice, design and evaluation of such interventions, especially in low and middle-income countries. Challenges include distal interventions, limits to the modifiability of some social and economic determinants, poorly understood mechanisms, difficulty defining the boundaries of such interventions, the need for inter-disciplinary and inter-sectoral collaboration, limited datasets in LMIC, sample size challenges for prevention interventions, ethical issues and siloed research funding. Potential approaches include the development of more robust causal models, trial designs that allow for analysis of mechanisms and the pooling of data across diverse settings to explore the role of contextual variables. Several criteria can inform the selection of interventions that target social determinants and these include the need for plausible mechanisms, feasibility, acceptability, cultural validity of moderator, mediator and outcome variables, generalizability and sustainability. These approaches require a high level of inter-sectoral and inter-disciplinary cooperation and data sharing across sites internationally. Examples are provided from ongoing research in LMIC.

## Highlights

• Social determinants play a vital role in shaping the mental health of populations.
• Despite many challenges, there are opportunities for intervening to address these social determinants.
• Several promising approaches can inform the choice, design and evaluation of interventions to address social determinants.
• This article presents some of these approaches, with illustrations from current research in low and middle-income countries.

## 1 Introduction

The field of global mental health was catalyzed through the publication of the first Lancet Series on Global Mental Health in 2007 (Lancet Global Mental Health Group, 2007). The final article in the series called for scaling up mental health care especially in low- and middle-income countries (LMIC) to address the massive treatment gap.

The field of global mental health has since diversified and expanded in several ways. Landmark publications such as the ‘Grand Challenges in Global Mental Health’ in 2011 have articulated a research agenda for the field, and this has been followed by significant new funding, particularly for research in LMIC (Collins et al., 2011). International agencies such as the World Health Organization (WHO) have supported the call for scaling up mental health services, largely through the flagship mhGAP programme, which now includes both psychological and pharmacological treatments and has been implemented in over 100 countries (WHO, 2016). WHO has also championed the human rights of people living with mental health conditions through its QualityRights programme, and advocacy for reform of mental health legislation (Funk & Bold, 2020; WHO, 2012). There has been a growing call to expand the focus from scaling up treatments, to also scaling up promotion and prevention interventions, including universal, selective and indicated prevention (Patel et al., 2018). There have also been robust debates regarding the meaning of the term ‘global’, and concerns expressed that the field of global mental health must critically engage with power and resource inequalities if it is to address global mental health disparities and develop partnerships based on principles of equity, freedom, diversity, agency and mutuality (Fekadu et al., 2021).

A key feature of this expansion and diversification has been a growing awareness of the importance of the social determinants of mental health. Increasing global income inequalities, poverty, food insecurity, gender-based violence and humanitarian emergencies caused by conflict and climate change are all powerful drivers of the mental health of populations (Lund et al., 2018). While the call to scale up mental health services remains relevant, the question articulated by Michael Marmot is equally salient: “why treat people only to send them back to the conditions that made them sick?” (Marmot, 2015).

This challenge of the social determinants of mental health is the focus of this article. I set out to present three main arguments. First, social determinants play a vital role in shaping the mental health of populations. Second, although there are major challenges, there are also important opportunities for intervening to address these social determinants. Third, there are several potential approaches and criteria which can inform the choice, design and evaluation of interventions to address the social determinants of mental health.

These arguments build on the evidence presented in the 2018 Lancet Commission on Global Mental Health and Sustainable Development, which demonstrated the importance of linking mental health with the broader human development agenda of the Sustainable Development Goals (SDGs) (Patel et al., 2018). This approach has become even more relevant in the COVID era, which has amplified the effects of known social determinants of mental health globally (Kola et al., 2021). It is well aligned with the broader burgeoning field of research and policy action on the social determinants of health, for example through the work of the WHO Commission on the Social Determinants of Health (WHO, 2008, 2010). It is also aligned with calls from more critical voices within the field of global mental health to focus on the social, structural and political determinants of mental health and move beyond narrow individualized treatments (Cosgrove et al., 2020).

## 2 What are social determinants of mental health?

Social determinants refer to the social and economic conditions that have a direct influence on the prevalence and severity of mental health conditions among females and males across the life course (Lund et al., 2018). This includes three important aspects: the structural social and economic arrangements which confer advantage or disadvantage on specific populations; differential exposure to adverse life events, which are related to social and economic position; and the specific conditions of vulnerability or resilience that these arrangements and exposures produce.

Social determinants of mental health can be organized into five main domains: the demographic, economic, neighborhood, environmental events, and social/cultural domains (Lund et al., 2018). Within each of these domains, social determinants exert their influence on the mental health of individuals across the life course, through a combination of distal and proximal factors. For example, within the economic domain, the distal impacts of economic recessions precipitated by the COVID pandemic has had an impact on depression outcomes in working age adults through its effect on more proximal experiences of financial insecurity, food insecurity, unemployment and eroded assets (Kola et al., 2021). Similarly, the distal effects of community violence may be experienced through bullying in school environments, which in turn is associated with adverse mental health outcomes for children and adolescents (Aisenberg & Herrenkohl, 2008; Albdour & Krouse, 2014). The evidence for the effects of social determinants on mental health in these five domains has been synthesized in a systematic review of 289 reviews and published previously (Lund et al., 2018). As the focus of this paper is on interventions to address these social determinants, this evidence will not be repeated here.

## 3 Intervening to address social determinants

The overwhelming evidence of the negative and interacting effects of social determinants on mental health begs the question: how can we go about intervening to address the social determinants of mental health? This is important not only from a clinical point of view, as a means of preventing mental health conditions. It is also vital for reasons of social justice. The massive inequalities in the distribution of wealth (with the richest 1% of the world's population earning twice as much as the rest of the world during the last 2 years (Christensen et al., 2023)), the looming reality of climate change, and ongoing humanitarian crises precipitated by war and conflict should not be allowed to continue, given the toll of human suffering they exact. In short, there are both instrinsic and instrumental reasons for addressing the social determinants of mental health: intrinsic because addressing social determinants such as poverty, climate change, conflict and child maltreatment are concerns of social justice; and instrumental because addressing these social determinants has the potential for large scale mental health benefits, particularly in vulnerable populations.

### 3.1 Candidate interventions

In a landmark meeting convened by the Academy of Medical Sciences and the InterAcademy partnership on the eve of the COVID pandemic in November 2019, representatives from 21 LMIC gathered to identify potential avenues for research and policy action to address the social determinants of mental health (Rose-Clarke et al., 2020). The InterAcademy partnership is a global network of science, engineering and medical academies that work together to provide independent expert advice for policy and practice. This meeting was one of a series of three meetings that the Academy has convened on the field of global mental health from 2018 to 2022. Using the framework of the five domains of the social determinants (described above), a set of intervention priorities were identified (See Box 1).

Box 1 **Examples of candidate interventions to address the social determinants of mental health, identified by the InterAcademy partnership meeting, 2019 (Rose-Clarke et al., 2020).**

•Demographic domain: interventions to reduce gender-based violence and child maltreatment
•Economic domain: social protection policies such as cash transfers
•Neighborhood domain: urban renewal and housing programmes
•Environmental events domain: improved disaster responses and mitigation of climate change
•Social and cultural domain: interventions to improve social capital and quality education

Alt-text: Box 1

Encouragingly, there is emerging evidence that some of these interventions can be effective, including in LMIC. For example, since the 1990s, conditional and unconditional cash transfers have increasingly been adopted by governments as a strategy for poverty reduction and human capital investment (Baird et al., 2014). A recent meta-analysis of the effects of cash transfers from 45 studies in LMIC demonstrated small but significant effect sizes (Cohen's d of 0.07 (95% confidence interval [CI] 0.05, 0.09) and 0.13 (95% CI 0.09, 0.18) on mental health and wellbeing outcomes respectively (McGuire et al., 2022).

When considering these interventions, an important distinction needs to be made between addressing social determinants in general populations (which may be construed as mental health promotion and prevention interventions, including universal, selective and indicated prevention); and addressing social determinants in clinical populations (which include addressing the social conditions of people living with mental health conditions). The former has been seen historically as the domain of international and community development and the latter as the domain of psychosocial rehabilitation. This article includes both types of interventions to address social determinants.

### 3.2 Challenges

Although there is promising evidence for the effectiveness of some interventions that address social determinants, when faced with the reality of designing and implementing these interventions, several challenges are evident, particularly in relation to mental health. First, these interventions are often quite distal to the mental health outcomes they hope to influence. Second, such interventions often require major social, economic and sometimes political changes which are very difficult to implement without substantial political will and resources. Third, the mechanisms by which such interventions yield improvements in the mental health outcomes of populations are currently poorly understood. Fourth, it may be difficult to determine the boundaries of interventions that target social determinants, as a broad range of social, economic and political challenges may be seen as social determinants of mental health in a given context. Fifth, the design and evaluation of these interventions requires interdisciplinary approaches and a high level of collaboration between research, policy and implementing partners. Sixth, in LMIC there are currently limited available data sets with culturally valid mental health outcome measures to evaluate these interventions. Seventh, sample size remains a major challenge in prevention trials as these studies may not be adequately powered to determine whether an identified social determinant reduces the incidence of a particular mental health outcome (Cuijpers, 2003). Eighth, there are significant ethical challenges with designing and implementing randomized controlled trials for these interventions. And finally, research in this field presents important challenges for research funders including the need to support interdisciplinary research and convene interdisciplinary review panels.

## 4 Potential approaches

Despite these challenges, there are some potential approaches to moving this field forward. These require three key steps.

### 4.1 Building theoretical models

First, it is important to build theoretical models which set out the hypothesized causal mechanisms by which an intervention that addresses social determinants will yield improvements in mental health outcomes. One such approach is the use of Theory of Change models, which draw on the evidence and opinions from a wide range of local stakeholders to articulate the steps along an hypothesized causal pathway to the intended impact (Breuer et al., 2016). For example if we are to test the effects of cash transfer programs on the prevention of depression among refugee youth, we need to identify the social and neuropsychological mechanisms which may mediate the effects of cash transfers on depression in this age group. This is an important departure from the use of Theory of Change in global mental health research in two respects: first, more robust data on these distal and proximal mechanisms are required to test the assumptions underlying interventions that address social determinants; and second, Theory of Change workshops comprising stakeholders from diverse disciplines and sectors need to be convened to collaboratively develop Theory of Change pathways that adequately address social determinants.

These examples of theory development refer largely to so-called ‘programme theory’ and ‘middle range theory’, which respectively attempt to develop explanatory models for how a specific intervention works in a specific context; and how these programme theories can generalize across diverse settings and still be tested (Shearn et al., 2017). Broader engagement with ‘grand theory’, such as Amartya Sen's human capabilities theory, is a crucial area of ongoing work to address the social determinants of mental health (Sen, 1999).

### 4.2 Testing mechanisms

**Fig. 1:**
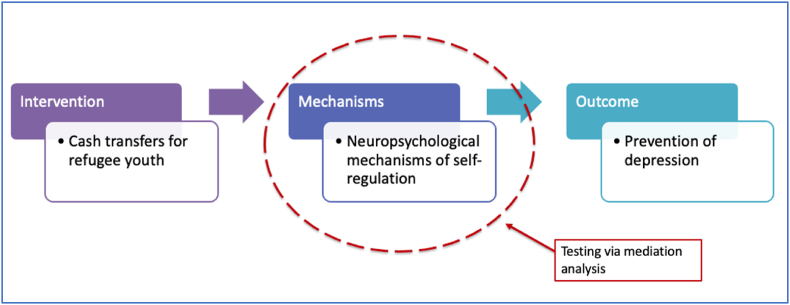
Testing specific mechanisms in hypothesized causal pathways of the social determinants of mental health: an example of cash transfers for refugee youth.

Second, having built our initial theoretical models, we need to test specific mechanisms in these models. For example, the design of randomized controlled trials to test these interventions must include mediation analysis to identify which variables mediate the effect of the intervention on the outcome. Improved understanding of these mechanisms may help us to fine tune and optimize interventions. To continue with the example of cash transfers among refugee youth, if we find reasons to believe that specific social mechanisms (such as peer support) or specific neuropsychological mechanisms (such as self-regulation) are important mediators of the effects of cash transfers on depression, then adjunct interventions that enhance these mechanisms (for example by building peer support, or teaching self-regulation skills) may potentiate the effects of cash transfers on depression (See Fig. 1).

This also extends to the examination of moderators and moderated mediation. For example, gender may be an important moderator of the impact of youth employment programmes on mental health outcomes. In the case of moderated mediation, the effect of a social determinant such as food insecurity on depression via a mediator variable such as self-regulation may be moderated by gender. Crucially, trials need to be adequately powered to assess mediation, moderation and moderated mediation.

Encouragingly, some work on testing the mechanisms of social determinants on mental health have already been undertaken in recent studies. For example, McGuire and colleagues’ meta-regression found that the value of the cash transfer, in both absolute terms and relative to previous income was a strong predictor of the effect on mental health outcomes (McGuire et al., 2022). This could be explored further in future studies examining the effect of varying the amount, timing and conditionality of cash transfers on mental health outcomes (Evans-Lacko et al., 2023).

### 4.3 Pooling data

Third, it is vital that we pool data across multiple sites to examine the effect of specific contextual variables on the intervention, mediators and outcomes. Realist evaluation methods are potentially helpful here, as they set out to identify the context and mechanisms by which specific interventions that target social determinants may influence mental health outcomes (so-called Context-Mechanisms-Outcomes or C-M-O models) (Pawson et al., 2005). For example, optimising an intervention to break cycles of unemployment and anxiety among youth in a particular setting in Latin America or sub-Saharan Africa may benefit from looking beyond traditional systematic reviews and meta-analyses to realist reviews. Realist reviews enable the identification of multiple C-M-O configurations that may explain the observed association between youth unemployment and anxiety, facilitating a more targeted approach that is responsive to local contexts.

### 4.4 Building consensus and interdisciplinarity

All of this requires a high level of consensus on plausible causal pathways and measures. It also requires a high level of cooperation and data sharing across sites internationally, given the importance of context in this field. And finally, it requires a high level of interdisciplinarity between fields such as economics, epidemiology, psychology, psychiatry and anthropology, with active participation by people with lived experience of mental health conditions in these settings. Until recently, the development of interventions in the field of global mental health has tended to remain siloed, for example within psychological treatments, cash transfer programmes, pharmacological treatments or education interventions, which tend to be evaluated in parallel lines of enquiry. Addressing the social determinants of mental health requires us to explore the added value of combined approaches from different sectors and disciplines.

### 4.5 Choosing which social determinants to target

This then raises the important question of which social determinants should be targeted by such interventions. Several criteria may be considered when making these choices. First, there should be a plausible mechanism linking the intervention on a specific social determinant to an identified mental health outcome. Second, the intervention should be acceptable and feasible to implement in the given setting. Third, there should be reliable and culturally valid moderator, mediator and outcome measures, particularly important for the diverse social and cultural contexts of global mental health. Fourth, there should be the potential for generalizability and population level impact if the intervention is to be scaled up. And finally, the intervention should be sustainable, which includes potential channels for future financing and the political will and governance systems for horizontal and vertical scale up and community and stakeholder buy-in. This latter criterion is only made possible through strong and meaningful community engagement and partnership from the outset.

### 4.6 Current examples

To illustrate, I draw on examples from two ongoing research projects working in this field in LMIC. In the CHANCES-6 study, we have examined the effects of cash transfer programs on the mental health and life chances of young people in Brazil, Colombia, Liberia, Malawi, Mexico and South Africa (Bauer et al., 2021). In doing so, we have identified several considerations which may improve the mental health impacts of cash transfer programmes in these countries. These include the context, conditionality, targeting and value of cash transfer programs, which all have the potential to influence the yield of mental health improvements in young people who receive these interventions (Evans-Lacko et al., 2023).

In the “Improving adolescent mental health by reducing the impact of poverty” (ALIVE) study, we are working in Colombia, Nepal and South Africa to design a selective prevention intervention for adolescent depression and anxiety (ALIVE, 2023). Our hypothesis is that multidimensional poverty increases risk for depression and anxiety among adolescents both directly and through it's impact on the capacity for self-regulation. This is a 4-arm pilot randomized controlled trial, with the arms comprising (1) an intervention to strengthen self-regulation; (2) an economic intervention which includes cash transfers, financial literacy, information on economic returns to completing education and negotiation skills; (3) a ‘combined’ intervention, including self-regulation and the economic intervention components; and (4) a control arm. We will set out to identify the feasibility and acceptability of delivering the interventions in contexts of urban poverty in Bogota, Cape Town and Kathmandu, while developing and validating key measures of eligibility, implementation, mediators and outcomes.

## 5 Policy challenges

Broadening the field of global mental health to more proactively addressing the social determinants of mental health brings a number of policy challenges. The first is the risk that policy messages expressed by mental health advocates become diluted. The advantage of the call to scale up mental health care is that this is a very focused issue, for which there is now a burgeoning evidence base and for which there are clearly identifiable costs, targets and indicators. Interventions to address social determinants are currently altogether more opaque in their mechanisms of action and in the evidence for their effectiveness.

Second, some may argue that advocacy messages regarding social determinants are not that different to the broader messages of the social determinants of health, or the Sustainable Development Goals (SDGs). Indeed, we have argued elsewhere that the task of addressing the social determinants of mental health is well aligned with the SDGs (Lund et al., 2018). Some might argue that if limited resources are to be allocated to the social determinants of mental health, these should focus on those social determinants for which mental health is particularly relevant, in a way that might not be the case for other health conditions. An example might be that of bullying, which may not be a major social determinant of health, but has been shown to be a powerful and growing predictor of poor mental health in the global burden of disease study (Hong et al., 2022).

A third policy challenge is the tradeoff of instrumental versus intrinsic arguments for addressing the social determinants of mental health. The instrumental argument is that addressing social determinants such as poverty and gender-based violence are important because they are a means of improving population mental health. But it can also be argued, as stated above, that poverty reduction and ending gender-based violence are worthy goals in their own right and have intrinsic value.

In a world of finite budgets, these policy challenges need to be addressed empirically, and the some of the tools for doing so are already available, as shown in the work of the Disease Control Priorities (DCP) and WHO-CHOICE (Patel et al., 2016). For example, comparative cost-effectiveness analyses can compare the cost-effectiveness of cash transfer programmes with scaling up behaviour activation therapy using task shared models for depression outcomes. An interesting example of this is a recent economic modeling study examining the return on investment and costs per Disability Adjusted Life Years (DALYs) averted for prevention and treatment interventions for depression, anxiety, bipolar disorder and suicide among adolescents (Stelmach et al., 2022). There is clearly a need for more economic evaluations, such as comparative cost-effectiveness of interventions that specifically address social determinants of mental health compared to providing treatments.

In relation to scaling up interventions that address social determinants of mental heath, there are also opportunities to build on the tools of implementation science, such as work undertaken to identify the mechanisms by which psychological treatments can be scaled up in low resource settings (Singla & Hollon, 2020).

## 6 Conclusions

Why is the consideration of social determinants important for behavioural science? Because it requires a clear delineation of the behavioural mechanisms by which social determinants influence mental health outcomes. Essential is the identification of targets for interventions that are behavioural (often in combination with structural interventions), to improve mental health outcomes. This is a potentially novel way of thinking about behavioural interventions by decoupling them from an exclusive focus on mental health outcomes, and instead thinking more carefully about the social aetiology of mental health conditions and aligning behavioural interventions accordingly.

For example, instead of focusing behavioural interventions exclusively on behaviours that reduce depression symptoms, the focus is broadened to include behaviours that change relationships with social drivers of depression (such as deprivation and threat). Or put another way, this requires an identification of the particular cognitive, behavioural and emotional patterns that are precipitated by living in circumstances of social or economic adversity, and developing interventions that attempt to change these patterns. This is well aligned with the growing field of prevention interventions, which emphasise the importance of acting on modifiable risk factors through universal, selective and indicated approaches (Fusar-Poli et al., 2021).

These investigations should not exclude enquiries into reverse causality. For example, there is growing evidence that psychological treatments yield economic benefits in LMIC. Seven-year follow-up of the landmark Thinking Healthy trial of maternal depression in Pakistan provided compelling evidence that a cognitive behaviour therapy intervention yielded sustained economic empowerment for mothers and increased time and money intensive parental investments by between 0.2 and 0.3 standard deviations (Baranov et al., 2020).

To conclude, an increased focus on the social determinants of global mental health identifies several opportunities for research, policy and practice. These include the need for inter-sectoral, inter-disciplinary ‘Whole-of-Society’ approaches, in which different arms of government and civil society collaborate to address both the causes and consequences of mental health conditions. For example, programmes to reduce gender-based violence, reduce income insecurity or improve food security need to examine their effects on the prevention of mental health conditions and the promotion of mental health and wellbeing. An example is so-called ‘cash-plus’ programmes which require intersectoral financing and inter-disciplinary approaches to evaluation (Little et al., 2021).

As the field of global mental health matures and diversifies, it is vital to sustain early calls to scale up mental health care, including through robust implementation science methods, while broadening the research, policy and practice agenda to address the social determinants of mental health. This paper has set out potential pathways for future development in this field.

## Declaration of competing interest

I declare that I have no conflicts of interest.

## Data Availability

No data was used for the research described in the article.

## References

[bib1] Aisenberg E., Herrenkohl T. (2008). Community violence in context: Risk and resilience in children and families. Journal of Interpersonal Violence.

[bib2] Albdour M., Krouse H.J. (2014). Bullying and victimization among african American adolescents: A literature review. Journal of Child and Adolescent Psychiatric Nursing.

[bib3] ALIVE (2023). Improving adolescent mental health by reducing the impact of poverty. https://www.alive4mentalhealth.org/.

[bib4] Baird S., Ferreira F.H.G., Özler B., Woolcock M. (2014). Conditional, unconditional and everything in between: A systematic review of the effects of cash transfer programmes on schooling outcomes. Journal of Development Effectiveness.

[bib5] Baranov V., Bhalotra S., Biroli P., Maselko J. (2020). Maternal depression, women's empowerment, and parental investment: Evidence from a randomized controlled trial. The American Economic Review.

[bib6] Bauer A., Baltra R.A., Pabon M.A., Diaz Y., Garman E., Hessel P., Lund C., Malvasi P., Matijasevich A., McDaid D., Park A.L., Paula C.S., Zimmerman A., Evans-Lacko S. (2021). Examining the dynamics between young people's mental health, poverty and life chances in six low- and middle-income countries: Protocol for the CHANCES-6 study. Social Psychiatry and Psychiatric Epidemiology.

[bib7] Breuer E., De Silva M.J., Shidaye R., Petersen I., Nakku J., Jordans M.J., Fekadu A., Lund C. (2016). Planning and evaluating mental health services in low- and middle-income countries using theory of change. British Journal of Psychiatry.

[bib8] Christensen M.-B., Hallum C., Maitland A., Parrinello Q., Putarato C., Abed D., Brown C.H., Kamande A., Lawson M., Ruiz S. (2023).

[bib9] Collins P.Y., Patel V., Joestl S., March D., Insel T.R., Daar A.S. (2011). Grand challenges in global mental health. Nature.

[bib10] Cosgrove L., Mills C., Karter J.M., Mehta A., Kalathil J. (2020). A critical review of the Lancet Commission on global mental health and sustainable development: Time for a paradigm change. Critical Public Health.

[bib11] Cuijpers P. (2003). Examining the effects of prevention programs on the incidence of new cases of mental disorders: The lack of statistical power. American Journal of Psychiatry.

[bib12] Evans-Lacko S., Araya R., Bauer A., Garman E., Alvarez-Iglesias A., McDaid D., Hessel P., Matijasevich A., Silvestre Paula C., Park A., Lund C. (2023). Potential mechanisms by which cash transfer programmes could improve the mental health and life chances of young people: A conceptual framework and lines of enquiry for research and policy. Global Mental Health.

[bib13] Fekadu A., Assefa E., Tesfaye A., Hanlon C., Adefris B., Manyazewal T., Newport M.J., Davey G. (2021). Towards effective and sustainable global academic partnerships through a maturity model informed by the capability approach. Global Health.

[bib14] Funk M., Bold N.D. (2020). WHO's QualityRights initiative: Transforming services and promoting rights in mental health. Health Human Rights.

[bib15] Fusar-Poli P., Correll C.U., Arango C., Berk M., Patel V., Ioannidis J.P.A. (2021). Preventive psychiatry: A blueprint for improving the mental health of young people. World Psychiatry.

[bib16] Hong C., Liu Z., Gao L., Jin Y., Shi J., Liang R., Maimaitiming M., Ning X., Luo Y. (2022). Global trends and regional differences in the burden of anxiety disorders and major depressive disorder attributed to bullying victimisation in 204 countries and territories, 1999-2019: An analysis of the global burden of disease study. Epidemiology and Psychiatric Sciences.

[bib17] Kola L., Kohrt B.A., Hanlon C., Naslund J.A., Sikander S., Balaji M., Benjet C., Cheung E.Y.L., Eaton J., Gonsalves P., Hailemariam M., Luitel N.P., Machado D.B., Misganaw E., Omigbodun O., Roberts T., Salisbury T.T., Shidhaye R., Sunkel C., Patel V. (2021). COVID-19 mental health impact and responses in low-income and middle-income countries: Reimagining global mental health. The Lancet Psychiatry.

[bib18] Lancet Global Mental Health Group (2007). Scale up services for mental disorders: A call for action. Lancet.

[bib19] Little M.T., Roelen K., Lange B.C.L., Steinert J.I., Yakubovich A.R., Cluver L., Humphreys D.K. (2021). Effectiveness of cash-plus programmes on early childhood outcomes compared to cash transfers alone: A systematic review and meta-analysis in low- and middle-income countries. PLoS Medicine.

[bib20] Lund C., Brooke-Sumner C., Baingana F., Baron E.C., Breuer E., Chandra P., Haushofer J., Herrman H., Jordans M., Kieling C., Medina-Mora M.E., Morgan E., Omigbodun O., Tol W., Patel V., Saxena S. (2018). Social determinants of mental disorders and the sustainable development goals: A systematic review of reviews. The Lancet Psychiatry.

[bib21] Marmot M. (2015).

[bib22] McGuire J., Kaiser C., Bach-Mortensen A.M. (2022). A systematic review and meta-analysis of the impact of cash transfers on subjective well-being and mental health in low- and middle-income countries. Nature Human Behaviour.

[bib23] Patel V., Chisholm D., Dua T., Laxminarayan R., Medina Mora M.E. (2016).

[bib24] Patel V., Saxena S., Lund C., Thornicroft G., Baingana F., Bolton P., Chisholm D., Collins P.Y., Cooper J.L., Eaton J., Herrman H., Herzallah M.M., Huang Y., Jordans M.J.D., Kleinman A., Medina-Mora M.E., Morgan E., Niaz U., Omigbodun O., UnUtzer J. (2018). The Lancet Commission on global mental health and sustainable development. Lancet.

[bib25] Pawson R., Greenhalgh T., Harvey G., Walshe K. (2005). Realist review--a new method of systematic review designed for complex policy interventions. Journal of Health Services Research & Policy.

[bib26] Rose-Clarke K., Gurung D., Brooke-Sumner C., Burgess R., Burns J., Kakuma R., Kusi-Mensah K., Ladrido-Ignacio L., Maulik P.K., Roberts T., Walker I.F., Williams S., Yaro P., Thornicroft G., Lund C. (2020). Rethinking research on the social determinants of global mental health. The Lancet Psychiatry.

[bib27] Sen A. (1999).

[bib28] Shearn K., Allmark P., Piercy H., Hirst J. (2017). Building realist program theory for large complex and messy interventions. International Journal of Qualitative Methods.

[bib29] Singla D.R., Hollon S.D. (2020). The importance of implementation science in scaling up psychological treatments. Behaviour Research and Therapy.

[bib30] Stelmach R., Kocher E.L., Kataria I., Jackson-Morris A.M., Saxena S., Nugent R. (2022). The global return on investment from preventing and treating adolescent mental disorders and suicide: A modelling study. BMJ Global Health.

[bib31] Who (2008).

[bib32] Who (2010). https://apps.who.int/iris/handle/10665/44488.

[bib33] Who (2012).

[bib34] Who (2016).

